# LncRNA-ATB participates in the regulation of calcium oxalate crystal-induced renal injury by sponging the miR-200 family

**DOI:** 10.1186/s10020-021-00403-2

**Published:** 2021-11-04

**Authors:** Yinhui Li, Tao Ding, Haiyan Hu, Tingting Zhao, Chao Zhu, Jiarong Ding, Jihang Yuan, Zhiyong Guo

**Affiliations:** 1 Department of Nephrology, The First Affiliated Hospital of Naval Military Medical University, Shanghai, 200433 People’s Republic of China; 2 Department of Medical Genetics, The Naval Military Medical University, Shanghai, 200433 People’s Republic of China

**Keywords:** lncRNA-ATB, EMT, miR-200a, Calcium oxalate monohydrate, Kidney stone, Calcium oxalate

## Abstract

**Background:**

LncRNA-ATB is a long noncoding RNA (lncRNA) activated by transforming growth factor β (TGF-β) and it has important biological functions in tumours and nontumour diseases. Meanwhile, TGF-β is the most critical regulatory factor in the process of nephrotic fibrosis and calcium oxalate (CaOx) crystal-induced renal injury. The present study aimed to investigate the biological function and mechanism of lncRNA-ATB in CaOx crystal-induced renal injury.

**Methods:**

The expression level of lncRNA-ATB was detected by quantitative reverse-transcription polymerase chain reaction (qRT-PCR), the expression levels of epithelial-mesenchymal transition (EMT) markers, TGF-β1 and Kidney Injury Molecule-1 (KIM-1) were detected by qRT-PCR, immunofluorescence staining or western blot analysis, cell proliferation was measured with a CCK-8 kit, cell apoptosis was measured by flow cytometry and TUNEL staining, and cell injury was detected with the Cytotoxicity lactate dehydrogenase (LDH) Assay kit and the expression level of KIM-1.

**Results:**

The expression levels of lncRNA-ATB and TGF-β1 were significantly increased in HK-2 cells after coincubation with calcium oxalate monohydrate (COM). COM stimulation caused significant injury in the HK-2 cells, induced cell apoptosis, inhibited cell proliferation, and induced EMT changes. After COM stimulation, the expression levels of the epithelial cell markers E-cadherin and zonula occludens (ZO)-1 in HK-2 cells significantly decreased, whereas the levels of the mesenchymal cell markers N-cadherin, vimentin and α-smooth muscle actin (α-SMA) significantly increased. Interference with lncRNA-ATB expression significantly relieved the COM-induced cell injury, cell apoptosis, proliferation inhibition, and EMT changes. The expression levels of the microRNA-200 (miR-200) family in the HK-2 cells after coincubation with COM were significantly decreased. MiR-200a mimics relieved the COM-induced cell injury, apoptosis, proliferation inhibition, and EMT changes, whereas miR-200a inhibitors abolished the lncRNA-ATB interference-induced relief of the COM-induced cell injury, apoptosis, proliferation inhibition, and EMT.

**Conclusion:**

LncRNA-ATB promoted the COM-induced cell injury, cell apoptosis, proliferation inhibition, and EMT to participate in the process of CaOx crystal-induced renal injury by sponging miR-200s.

**Supplementary Information:**

The online version contains supplementary material available at 10.1186/s10020-021-00403-2.

## Background

Kidney stones are a kind of common ailment and frequently encountered disease of the urinary system worldwide. Its annual prevalence worldwide is 3–5% and the lifetime prevalence of humans is 15–25% (Bigoniya and Sohgaura 2017). It has become an important risk factor for chronic kidney disease (CKD) and end-stage renal disease (ESRD) (Rule et al. 2009). Although the advancement of minimally invasive surgical technology has allowed great progress in the surgical treatment of stones, statistical analysis has shown that the recurrence rate of stones is 50% over 5–10 years and 75% over 20 years period after the first treatment (Bigoniya and Sohgaura 2017). Analysis of the components of urinary tract stones has shown that the major component of stones in more than 80% of patients is calcium oxalate (CaOx), which is mainly calcium oxalate monohydrate (COM) (Yasui et al. 2008, 2017). Kidney crystals are the early stage of stones. Numerous crystals form in the urine of normal people. Under normal conditions, the retention time of crystals in renal tubules is too short for them to grow big enough to block renal tubules. Crystals may adhere to renal tubular epithelial cells and stay in the kidney only when tubular epithelial cells are injured, and then gradually develop into stones. Therefore, renal tubular epithelial cell injury is considered the premise and starting point of stone formation (Khaskhali et al. 2009; Hirose et al. 2010; Rodgers 2017).

Long noncoding RNAs (lncRNA) are RNAs that are longer than 200 bp and lack protein-coding ability, but they still have important biological functions in tumours and nontumour diseases. Some of them play important regulatory roles in urinary tract calculus and CKD (Song et al. 2019; Zhou et al. 2019; Chen et al. 2020). LncRNA-ATB is a lncRNA activated by transforming growth factor β (TGF-β). Previous study showed it promoted zinc finger E-box binding homeobox (ZEB) 1/2 expression by competitively sponging the microRNA-200 (miR-200) family to further regulate the process of epithelial-mesenchymal transition (EMT) (Yuan et al. 2014). TGF-β is the most critical regulatory factor in the process of nephrotic fibrosis (Meng et al. 2016) and also plays a key role in the process of CaOx crystal-induced renal injury (Convento et al. 2017; Liu et al. 2020b). However, the function of lncRNA-ATB in crystal-induced renal injury is still not clear. Therefore, the aim of this study was to investigate the biological function and the mechanism of action of lncRNA-ATB in CaOx crystal-induced renal injury.

## Materials and methods

### Cell culture

Human proximal tubular epithelial (HK‐2) cells were purchased from the American Type Culture Collection (ATCC, MD,USA) and cultured in DMEM/F12 (HyClone, Utah, USA) containing 10% foetal bovine serum (FBS, Gibco, New York, United States), 100 U/mL penicillin and 100 µg/mL streptomycin (Beyotime, Haimen, China) in 5% carbon dioxide at a temperature of 37 °C. COM crystals(Sigma-Aldrich, Missouri, USA) were baked in a 180 °C oven overnight for sterilization, added to DMEM/F12 culture medium, and stirred for 2 h using a magnetic stirrer which sterilized by the same method to prepare suspensions at specific concentrations. The crystal-induced renal injury and EMT cell model was established using COM suspension or culture medium containing TGF-β1 (10 ng/mL) (PeproTech, New Jersey, USA) as a replacement for the normal culture medium for a certain time. The miR-200a mimics and inhibitors were synthesized by Shanghai GenePharma Co., Ltd, China. Transfection was performed using Lipofectamine 3000 (Invitrogen, California, USA), and further experiments were performed after 6 h of transfection.

### Establishment of stable lncRNA-ATB-interfering cell Lines

The lncRNA-ATB-interfering lentivirus and the control lentivirus were constructed by Shanghai GenePharma Co., Ltd. The interference sequences are shown in Table 1. To obtain stably transfected cell lines, HK-2 cells were infected with the corresponding lentiviruses. After 48 h, successfully transfected cells were screened using puromycin (6 μg/mL). Identification and expansion of culture were performed after 2 weeks of screening.

**Table 1:** Primers used for shRNAs sequences

Names	Sequences5'-3'
shRNA-ATB-1 sense shRNA-ATB-1 anti-sense	GATCCGCCTTATGGCCTAGATTACCTTTCCATTCAAGAGATGGAAAGGTAATCTAGGCCATAAGGCTTTTTTG AATTCAAAAAAGCCTTATGGCCTAGATTACCTTTCCATCTCTTGAATGGAAAGGTAATCTAGGCCATAAGGCG T
shRNA-ATB-2 sense shRNA-ATB-2 anti-sense	GATCCGCCTGTCTGTATTTGCGAATACCTTTTTCAAGAGAAAAGGTATTCGCAAATACAGACAGGCTTTTTTG AATTCAAAAAAGCCTGTCTGTATTTGCGAATACCTTTTCTCTTGAAAAAGGTATTCGCAAATACAGACAGGCG
shRNA-NC sense shRNA-NC anti-sense	GATCCGTTCTCCGAACGTGTCACGTTTCAAGAGAACGTGACACGTTCGGAGAACTTTTTTG AATTCAAAAAAGTTCTCCGAACGTGTCACGTTCTCTTGAAACGTGACACGTTCGGAGAACG

### Quantitative reverse transcription-polymerase chain reaction (qRT-PCR)

Total cellular RNA was extracted by the Trizol (Invitrogen, California, USA) method. cDNA was synthesized with three-step reverse transcription with the Moloney murine leukaemia virus (M-MLV, Invitrogen, California, USA) reverse transcriptase. Preparation of cDNAs for quantitative detection of lncRNA-ATB and miR-200 s used specific reverse transcription primers to replace random primers. The reverse transcription primer sequences are shown in Additional file 1: Table S1. PCR amplification was performed using the SYBR Green system (Takara, Dalian, China) in an Applied Biosystems thermal cycler with StepOnePlus™ RT‐PCR System (ThermoFisher, MA, USA). The manipulation was performed strictly according to the instructions. Relative quantification was done using the 2^−ΔΔCT^ method. All primer sequences for qRT-PCR are listed in Additional file 1: Table S2.

### Western bloting analysis

HK-2 cells after the interventions above were washed with phosphate-buffered saline (PBS) and lysed and denatured with RIPA buffer on ice to prepare the sample solution. Protein electrophoresis was performed in precast gels (10%) (EpiZyme, Shanghai, China). Proteins were transferred onto a polyvinylidene fluoride (PVDF) membrane at 200 mA constant current. The membrane was blocked in protein-free rapid blocking solution at room temperature for 15 min and incubated with the primary antibodies specific for N-cadherin (ab18203, Abcam, Hong Kong, China), zonula occludens (ZO)-1 (ab61357, Abcam, Hong Kong, China), E-cadherin (3195, Cell Signaling, MA, USA), Vimentin (3390, Cell Signaling, MA, USA), TGF-β1 (ab92486, Abcam, Hong Kong, China), Smad3 (PAC123Mu01, Cloud-Clone Corp. Wuhan, China), p-Smad3 (S423/425, Cell Signaling, MA, USA) or GAPDH (MAB374, Merck, Darmstadt, Germany) at 4 °C on a shaker overnight. The membrane was washed and incubated with the secondary antibody at room temperature in the dark on a shaker for 1–2 h. Then, the membrane was washed again and scanned on an Odyssey scanner (Lincoln, NE, USA) to analyse the grey density values of protein bands. The experiment was repeated three times.

### Cell proliferation experiment (CCK-8)

All stably transfected cells were inoculated into 96-well plates at 8000 cells/well. On the next day, the culture medium was replaced with culture medium with or without COM (200 μg/mL). After cells were cultured for specific times, 10 µL of the CCK-8 working solution (Dojindo, Kyushu, Japan) was added into each well, and the culture was continued for another 2 h. The optical density (OD) values of all the cells were measured at 450 nm in a microplate multimode reader (BioTek, Vermont, USA). The percentage of cell proliferation was calculated according to the instructions provided by the reagent kit.

### Cytotoxicity lactate dehydrogenase (LDH) detection

The cell injury was assessed by a Cytotoxicity LDH Assay Kit‐WST (Dojindo, Kyushu, Japan). Briefly, each type of stably transfected cells were divided into three groups: A: experimental group (cell stimulated by COM); B: high control group (cells + lysis buffer); and C: low control group (cells without any specific treatment). Background control wells were set up in each group. Cells were inoculated into 96-well plates at 8000 cells/well. The culture medium was replaced on the next day with the culture medium with/without COM (200 μg/mL) and cells were cultured for another 48 h. Then 10 μL lysis buffer was added to the high control wells and incubated in a culture incubator for 30 min. Next, 100 μL working solution was added into each well, followed by incubation for 20 min. Finally, 50 μL termination solution was added to each well, and the OD values at 490 nm were measured immediately in a BioTek microplate reader. The OD value of the background control well was subtracted from the actual OD value in each group. The percentage of injured cells caused by COM was calculated using the following formula: ${\text{Cytotoxicity }}\left( \% \right)\, = \,\left( {{\text{A}}\, - \,{\text{C}}} \right)/\left( {{\text{B}}\, - \,{\text{C}}} \right)\, \times \,{1}00$.

### Detection of cell apoptosis by flow cytometry

Different cells lines were inoculated into six-well plates at 1.5 × 10^6^ cells/well. On the next day, the culture medium was replaced with the culture medium with or without COM. All cells from each well were collected after 48 h (including dead cells in the culture medium), resuspended in 200 µL cold PBS, and filtered through a sieve to prevent clogging of the flow cytometer by COM crystals. Next, 300 µL binding buffer was added and mixed thoroughly with 5 µL Annexin V-FITC and 5 µL propidium iodide (PI) (BD Biosciences, New Jersey, USA). The reaction was conducted in the dark for 15 min, and cells were detected by a flow cytometer (BD FACSCalibur, New Jersey, USA). The results were analysed in FlowJo software.

### Apoptosis detection by TdT-mediated dUTP Nick-End Labeling (TUNEL) assay

The cells were seeded in 24-well plates at 3 × 10^5^ cells per well, and the attached cells were incubated for 24 h; then, the medium was replaced with medium with or without COM for further culture for 48 h. The 24-well plates were removed, the medium was discarded, and the cells were washed once with PBS and then fixed in 4% paraformaldehyde for 30 min. The cells were washed again and incubated with PBS containing 0.3% Triton X-100 for 10 min at room temperature. After two washes, 50 µL of fluorescent labelled TUNEL assay solution (Beyotime Biotechnology, Shanghai, China) containing terminal deoxynucleotidyl transferase enzyme were added, and the samples were placed in a wet box and incubated in the dark for 60 min at 37 °C. Then, the cells were washed with PBS three times, antifluorescence quenching blocking solution (Beyotime Biotechnology, Shanghai, China) containing DAPI was added, and finally, the cells were observed and photographed under an inverted fluorescence microscope (Olympus, Tokyo, Japan).

### Immunofluorescence staining

The cell lines were seeded in 24-well plates at 3 × 10^5^ cells per well, and the attached cells were incubated for 24 h. After further treatment with TGF-β1 or COM for 48 h, the 24-well plates were removed and washed once with PBS, and the cells were fixed with 4% paraformaldehyde for 15 min. Then, the cells were washed three times with ice-cold PBS and incubated with PBS containing 0.3% Triton X-100 for 10 min at room temperature. The cells were washed again for 5 min three times with ice-cold PBS, blocked with 5% BSA for 1 h and incubated overnight at 4 °C with α-smooth muscle actin (α-SMA) (1:200, 48938S, CST, MA, USA), E-cadherin (1:200, 3195, CST, MA, USA) and KIM-1 (1:100, PAA785Mu01, Cloud-Clone Corp. Wuhan, China) primary antibodies. The cells were then washed for 5 min three times with ice-cold PBS and then incubated with an Alexa 488-labelled murine secondary antibody and CY3-labelled rabbit secondary antibody for 1 h at room temperature in the dark. Finally, an antifluorescence quenching blocking solution (Beyotime Biotechnology, Shanghai, China) containing DAPI was added, and the cells were observed and photographed under an inverted fluorescence microscope (Olympus, Tokyo, Japan).

### Dual-luciferase reporter experiment

Different cell lines were inoculated into six-well plates at 1.5 × 10^6^ cells/well for adherent culture for 24 h. The pGL3-ATB WT and pGL3-ATB MT plasmids carrying the firefly luciferase gene, the *Renilla* luciferase plasmid, and the miR-200a or miR-NC plasmid were transfected using Lipofectamine 3000. Plasmids and miRNA were constructed by Shanghai GenePharma Co., Ltd. After cotransfection of nucleic acids for 48 h, cells were collected, and changes in OD values were detected using the dual-luciferase reporter detection system (Promega, Madison, USA) in a microplate reader.

### Statistical analysis

All data are expressed as the mean ± standard deviation. Comparison between multiple groups was performed using one-way analysis of variance. The pairwise comparison between groups was performed using the least significant differences *t* (LSD-t) test when variances were homogeneous and Dunnett’s T3 test when the variances were not homogeneous. Two groups were compared with the two-tailed Student’s *t* test. *P* < 0.05 indicated that the difference had statistical significance. All obtained data were statistically analysed with SPSS21.0 software (IBM, New York, USA).

## Results

### Significantly high expression of lncRNA-ATB and TGF-β1 in the CaOx crystal-induced renal injury cell model

**Fig. 1:**
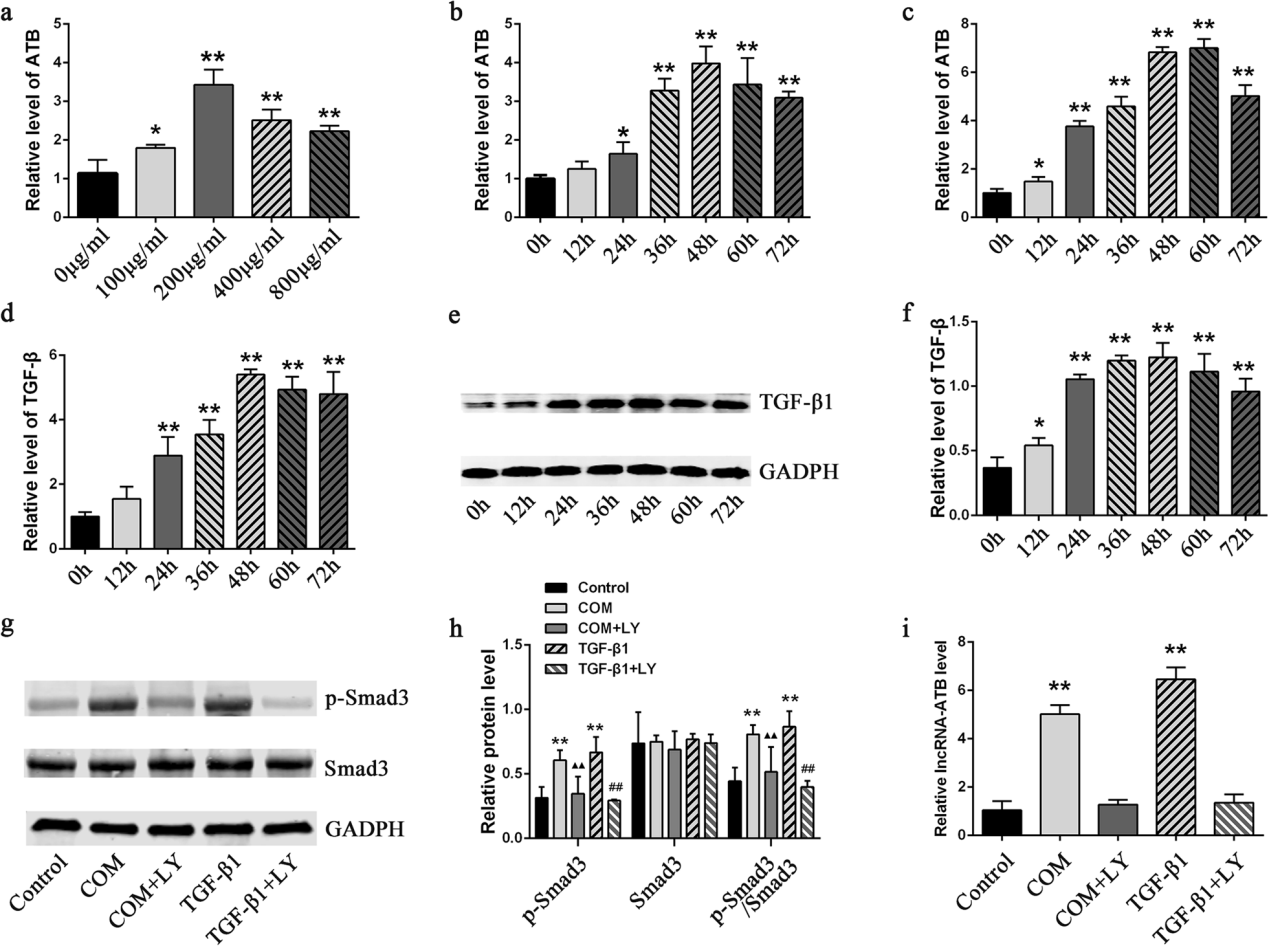
The expression levels of lncRNA-ATB and TGF-β1 in cell models. **a**–**c** LncRNA-ATB expression levels in HK-2 cells exposed to different-concentration COM for 48 h (**a**), to 200ug/ml COM for different hours (**b**), and to 10 ng/ml TGF-β1 for different hours **c** by qRT-PCR. **d**, **e** TGF-β1 expression levels in HK-2 cells exposed to 200ug/ml COM for different hours by qRT-PCR **d** and by Western bolting (**e**), **f** Quantitative results of TGF-β1 protein levels. **g** The Smad3 phosphorylation levels were detected by Western bolting. **h** Quantitative results of p-Smad3 or Smad3 expression levels. **i** LncRNA-ATB expression levels in HK-2 cells after the inhibition of the TGF-β1 receptor by LY2109761 All above the experiments were repeated 3 times, values are mean ± SD. *P < 0.05, **P < 0.01 *VS*. 0ug/ml or 0H or control group, ^▲▲^*P* < 0.01 vs. COM group, ^##^*P* < 0.01 VS. TGF-β1 group

Coincubation of HK-2 cells with COM is currently the major cell model of CaOx stone. We detected the expression levels of lncRNA-ATB in HK-2 cells with different COM concentrations and at different stimulation time points. The results showed that COM stimulation significantly increased the expression level of lncRNA-ATB in HK-2 cells in a dose- and time-dependent manner. Its expression level reached the peak value at the concentration of 200 μg/mL and the incubation time of 48 h (Fig. 1a, b). Application of TGF-β1 stimulation also significantly increased the expression level of lncRNA-ATB in HK-2 cells (Fig. 1c). In addition, detection of the TGF-β1 expression levels in HK-2 cells at different time points under 200 μg/mL COM stimulation showed that TGF-β1 was highly expressed in a time-dependent manner (Fig. 1d–f). We further detected the activation of the TGF-β/Smad3 pathway by performing a western blot analysis. The results showed that the SMAD3 phosphorylation levels were significantly increased in the HK-2 cells co-incubated with 200 μg/mL COM and 10 ng/mL TGF-β1 for 48 h, while the TGF-β1 receptor inhibitor LY2109761 (LY) significantly inhibited the phosphorylation of SMAD3 (Fig. 1g, h). In addition, the abnormally high expression of COM and TGF-β1-induced lncRNA-ATB in the HK-2 cells was reversed after the inhibition of the TGF-β1 receptor by LY2109761 (Fig. 1i).

### Significant EMT changes in HK-2 cells after COM and TGF-β1 stimulation

**Fig. 2:**
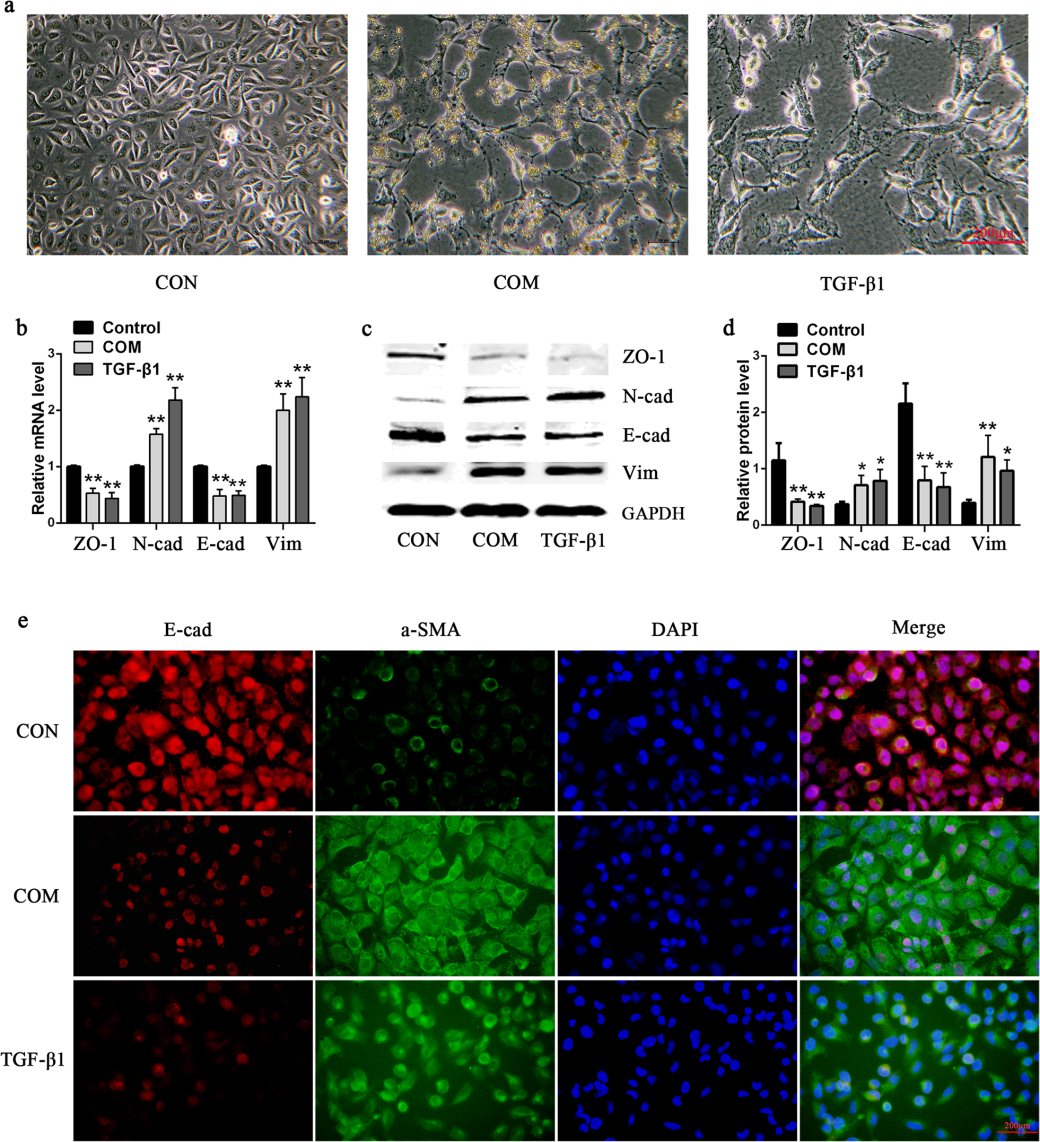
Significant EMT changes of HK-2 cells exposed to COM or TGF-β1. **a** Morphological Changes of HK-2 cells exposed to 200ug/ml COM or 10 ng/ml TGF-β1 for 48 h under light microscope. **b**, **c** The mRNA **b** or protein **c** levels of EMT markers in HK-2 cells exposed to 200ug/ml COM or 10 ng/ml TGF-β1 for 48 h. **d** Quantitative results of EMT marker-protein levels. **e** The expression levels of E-cadherin and α-SMA were detected by immunofluorescence. All above the experiments were repeated 3 times, values are mean ± SD. *P < 0.05, **P < 0.01 *VS*. Control group. Scale bars = 200 μm

After using COM or TGF-β1 to stimulate HK-2 cells for 48 h, the original cobblestone-like epithelial cell morphology of HK-2 cells was lost and was replaced by the spindle-shaped mesenchymal cell morphology. The tentacles of the cells grew in number and length, with an octopus-like pattern, and the number of cells decreased significantly (Fig. 2a). qRT-PCR and western bloting detection results showed that cell epithelial markers E-cadherin (E-cad) and ZO-1 significantly decreased and mesenchymal markers N-cadherin (N-cad) and vimentin (Vim) significantly increased (Fig. 2b–d). The changes in the EMT markers were further detected by immunofluorescence. The results showed that the E-cadherin expression levels were significantly decreased, while the α-SMA levels were significantly increased after the HK-2 cells were stimulated by COM or TGF-β1 (Fig. 2e).

### Relief of COM-induced cell injury, cell apoptosis, and proliferation inhibition by interference with lncRNA-ATB

**Fig. 3:**
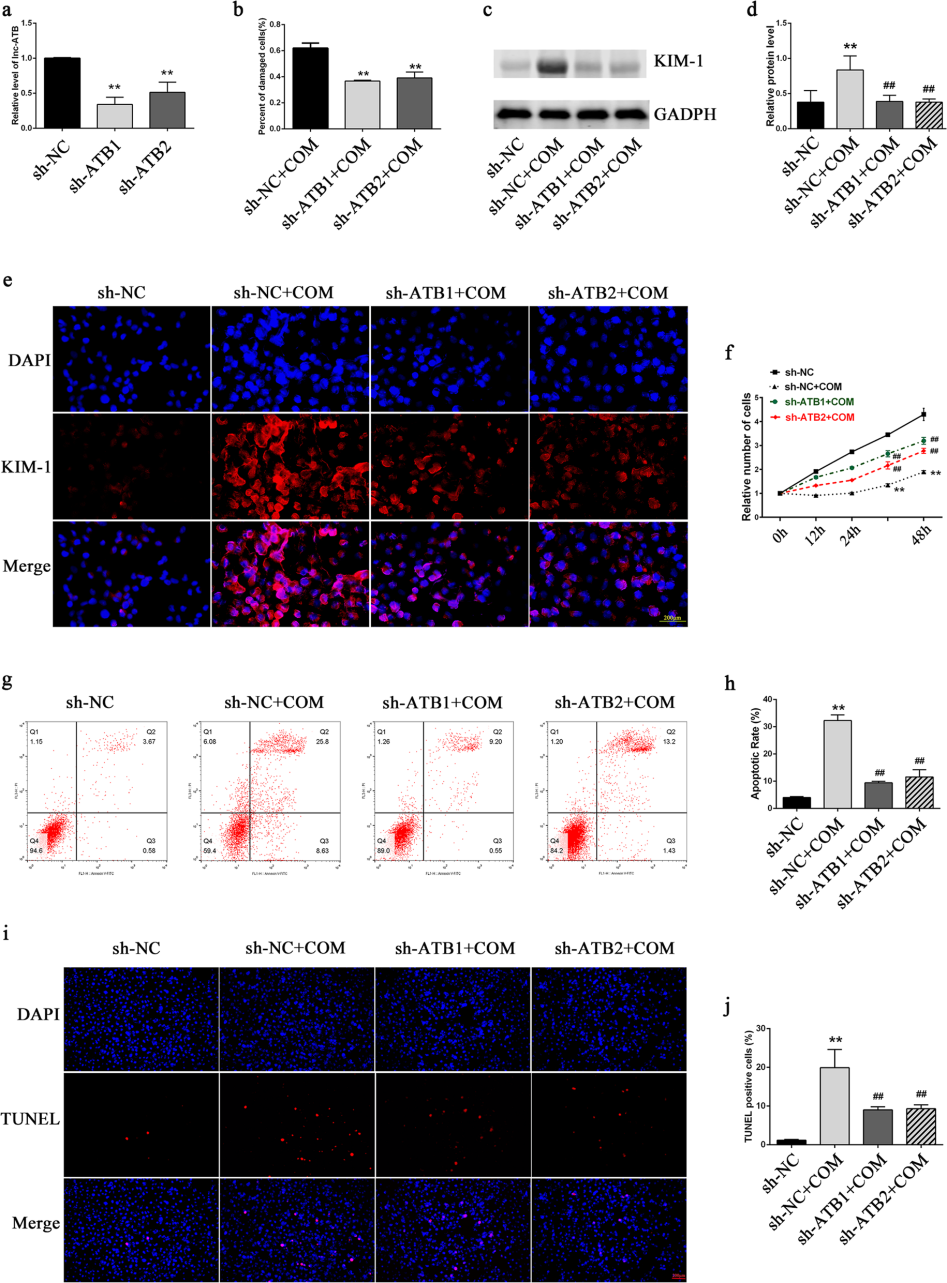
Functional effects of lncRNA-ATB knockdown in HK‐2 cells exposed to COM. **a** The interference efficiencies of interfering lentivirus were validated by qRT‐PCR. **b** Cell damage was assessed by a Cytotoxicity LDH Assay Kit. **c**, **d** The expression levels of KIM-1 were detected by western blot analysis. **e** The expression levels of KIM-1 were detected by immunofluorescence. **f** Cell proliferation was assayed by CCK-8. **g**–**k** Cell apoptosis was analysed by flow cytometry **g**, **h** and TUNEL staining (**i**, **j**). All above the experiments were repeated 3 times, values are mean ± SD; *P < 0.05, **P < 0.01 *VS*. sh-NC group; ^**##**^*P* < 0.01 *VS*. sh-NC + COM group. Scale bars = 200 μm

The stable lncRNA-TAB-knockdown cell line was established by transfecting interfering viruses into HK-2 cells. The interference efficiency was validated by qRT-PCR. The results showed that both interference sites significantly interfered with lncRNA-ATB expression (Fig. 3a). The western blot analysis and immunofluorescence staining of Kidney Injury Molecule-1 (KIM-1) and the LDH detection showed that interference with lncRNA-TAB expression significantly relieved the COM-induced cell injury (Fig. 3b–e). CCK-8 assay results showed that COM stimulation inhibited cell proliferation, while interference with lncRNA-ATB expression significantly relieved the COM-induced cell proliferation inhibition (Fig. 3f). Detection of cell apoptosis by flow cytometry and the TUNEL staining showed that interference with lncRNA-ATB expression significantly relieved the COM-induced cell apoptosis (Fig. 3g–j).

### Relief of COM-induced EMT changes in HK-2 cells by interference with lncRNA-ATB expression

**Fig. 4:**
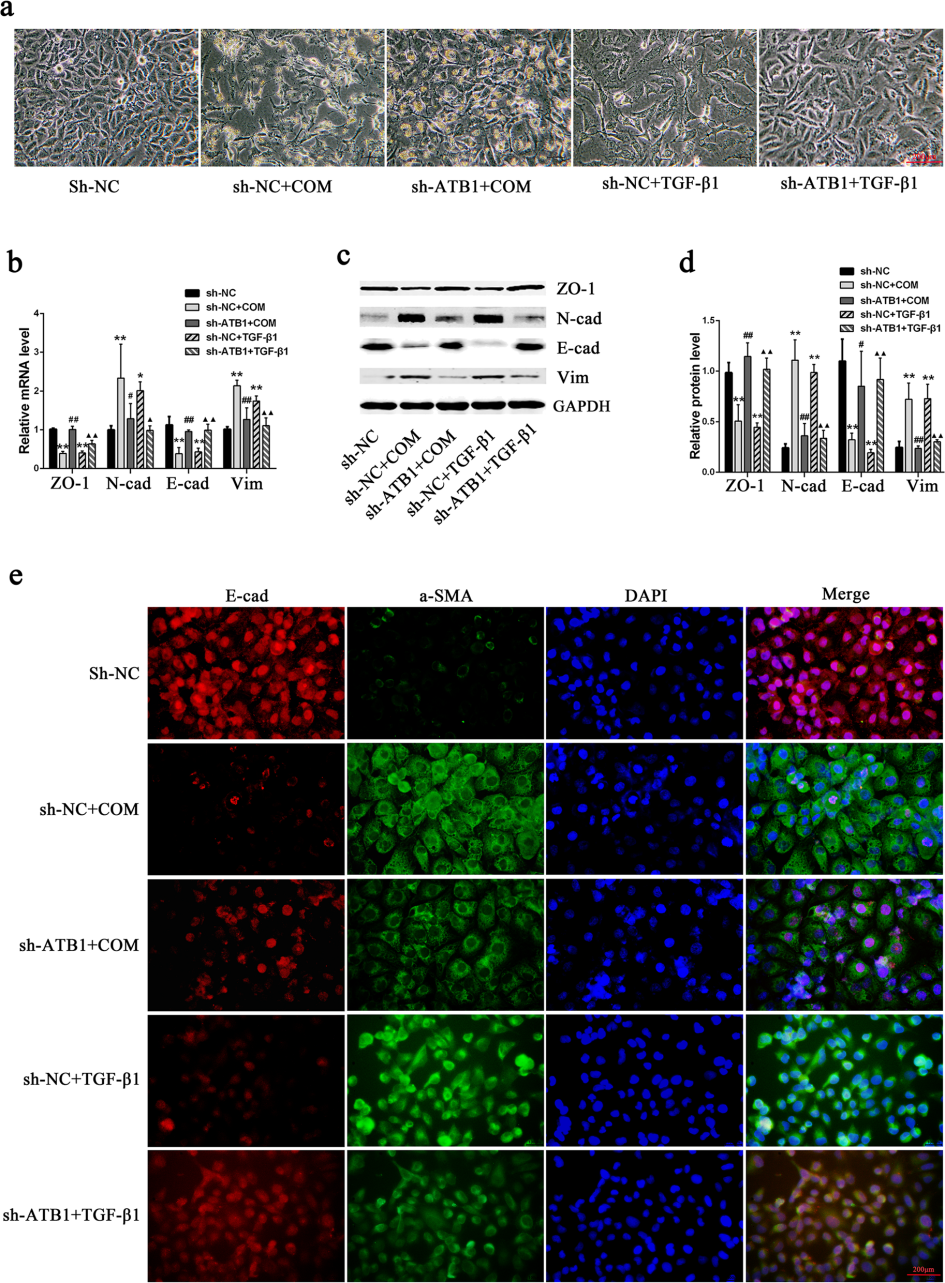
The effects of lncRNA-ATB knockdown on the COM-induced EMT in HK‐2 cells. **a** Morphological Changes of HK-2 cells exposed to 200ug/ml COM or 10 ng/ml TGF-β1 for 48 h under light microscope. **b,c** The mRNA **b** or protein **c** levels of EMT markers in HK-2 cells exposed to 200ug/ml COM or 10 ng/ml TGF-β1 for 48 h. **d** Quantitative results of EMT marker-protein levels. **e** The expression levels of E-cadherin and α-SMA were detected by immunofluorescence. All above the experiments were repeated 3 times, values are mean ± SD; *P < 0.05, **P < 0.01 VS. sh-NC group, ^#^*P* < 0.05, ^##^*P* < 0.01 VS. sh-NC + COM group, ^▲^*P* < 0.05, ^▲▲^*P* < 0.01 VS. sh-NC + TGF-β1 group, Scale bars = 200 μm

Observation of the effect of interference with lncRNA-ATB expression on COM-induced EMT in HK-2 cells showed that the COM-induced spindle shape and octopus-like mesenchymal cell morphology of HK-2 cells were significantly lessened, cell proliferation inhibition was significantly attenuated, and the cell number was significantly recovered (Fig. 4a). In addition, the above changes in epithelial cell markers E-cadherin and ZO-1 and mesenchymal markers N-cadherin and vimentin were significantly reversed (Fig. 4b–d). Similarly, the immunofluorescence results showed that the altered levels of E-cadherin and α-SMA expression were significantly reversed after interference with lncRNA-ATB expression (Fig. 4e).

### Targeted binding to lncRNA-ATB by the miR-200 family in HK-2 Cells

**Fig. 5:**
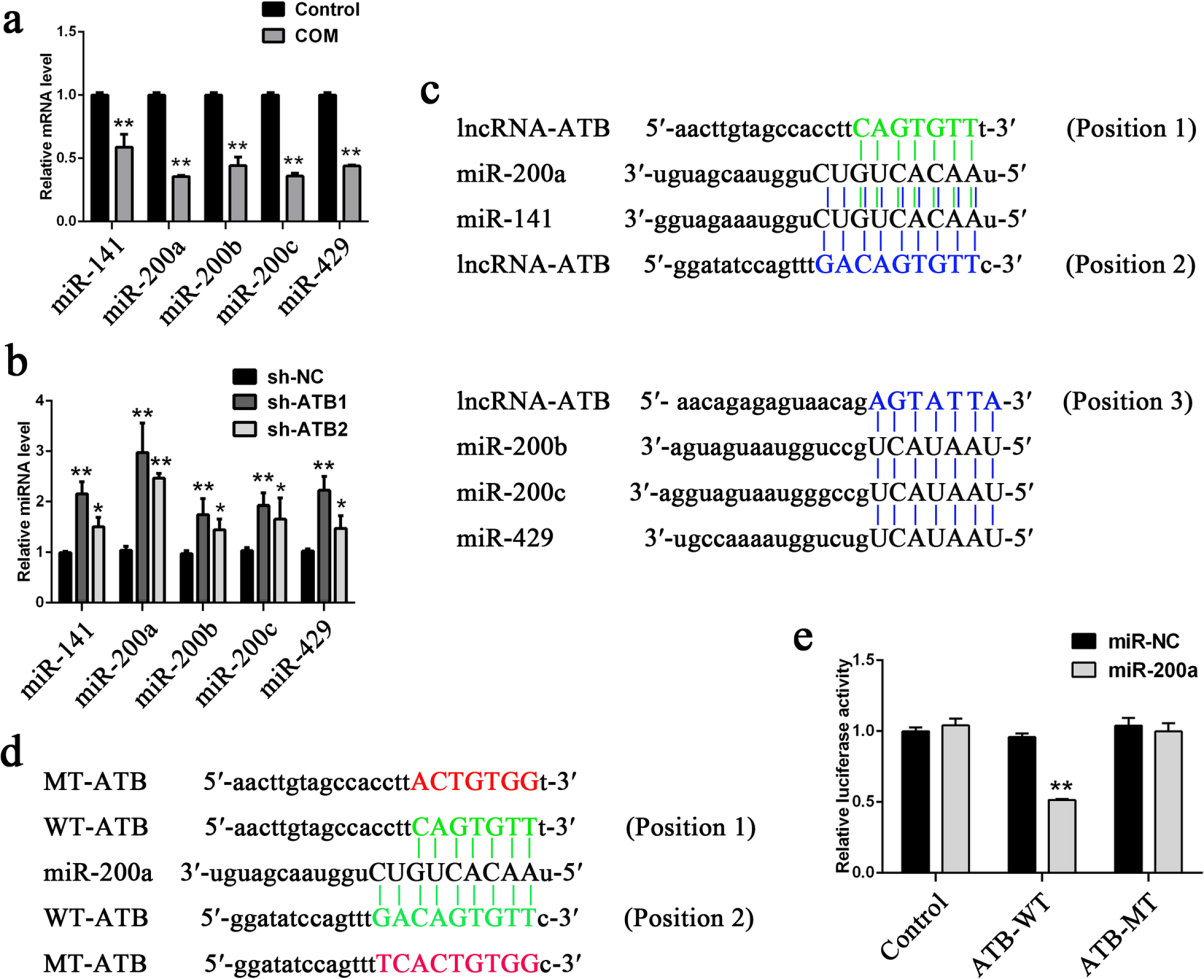
miR-200 family directly bind to lncRNA-ATB in HK-2 cells. **a**, **b** The expression levels of miR-200 family members **a** in HK-2 after COM stimulation and **b** in lncRNA-ATB knockdown cell lines. **c** The prediction for miR-200 family members’ binding sites on lncRNA-ATB transcript. **d** The binding sites of miR‐200a on lncRNA-ATB transcript are shown in green, and the mutant sequence is shown in red. **e** Dual‐luciferase reporter assays were performed using HK-2 cells cotransfected with miR-200a and luciferase reporters containing nothing, lncRNA-ATB wild-type (WT) or mutant transcript (MT). All above the experiments were repeated 3 times, values are mean ± SD; *P < 0.05, **P < 0.01 *VS*. Control, sh-NC or miR-NC group

In a previous study, we found that the miR-200 family could specifically bind to lncRNA-ATB to reverse lncRNA-ATB overexpression-induced EMT^[11]^. Here, the expression levels of the miR-200 family in the COM stimulation HK-2 cell model were detected, and the results showed that all members of the miR-200 family, including miR-141, miR-200a, miR-200b, miR-200c, and miR-429, had significantly lower expression in HK-2 cells with COM than without (Fig. 5a). Further detection of the expression levels of the miR-200 family in the two stably transfected cell lines showed that the expression levels of the miR-200 family significantly increased after lncRNA-ATB interference (Fig. 5b). To confirm whether lncRNA-ATB exerted its biological functions through the miR-200 family, the miR-200 member that had the most significantly differential expression, miR-200a, was selected for biological function rescue experiments. The miR-200 family-lncRNA-ATB binding sites were first predicted using bioinformatics technology (Fig. 5c). Next, whether miR-200a could directly interact with lncRNA-ATB was examined in dual luciferase reporter experiments. The results showed that miR-200a significantly decreased the luciferase intensity of lncRNA-ATB WT but did not decrease the luciferase intensity of lncRNA-ATB MT (Fig. 5d, e).

### Promotion of COM-induced cell injury, proliferation inhibition, cell apoptosis, and EMT changes by lncRNA-ATB through sponging miR-200a

**Fig. 6:**
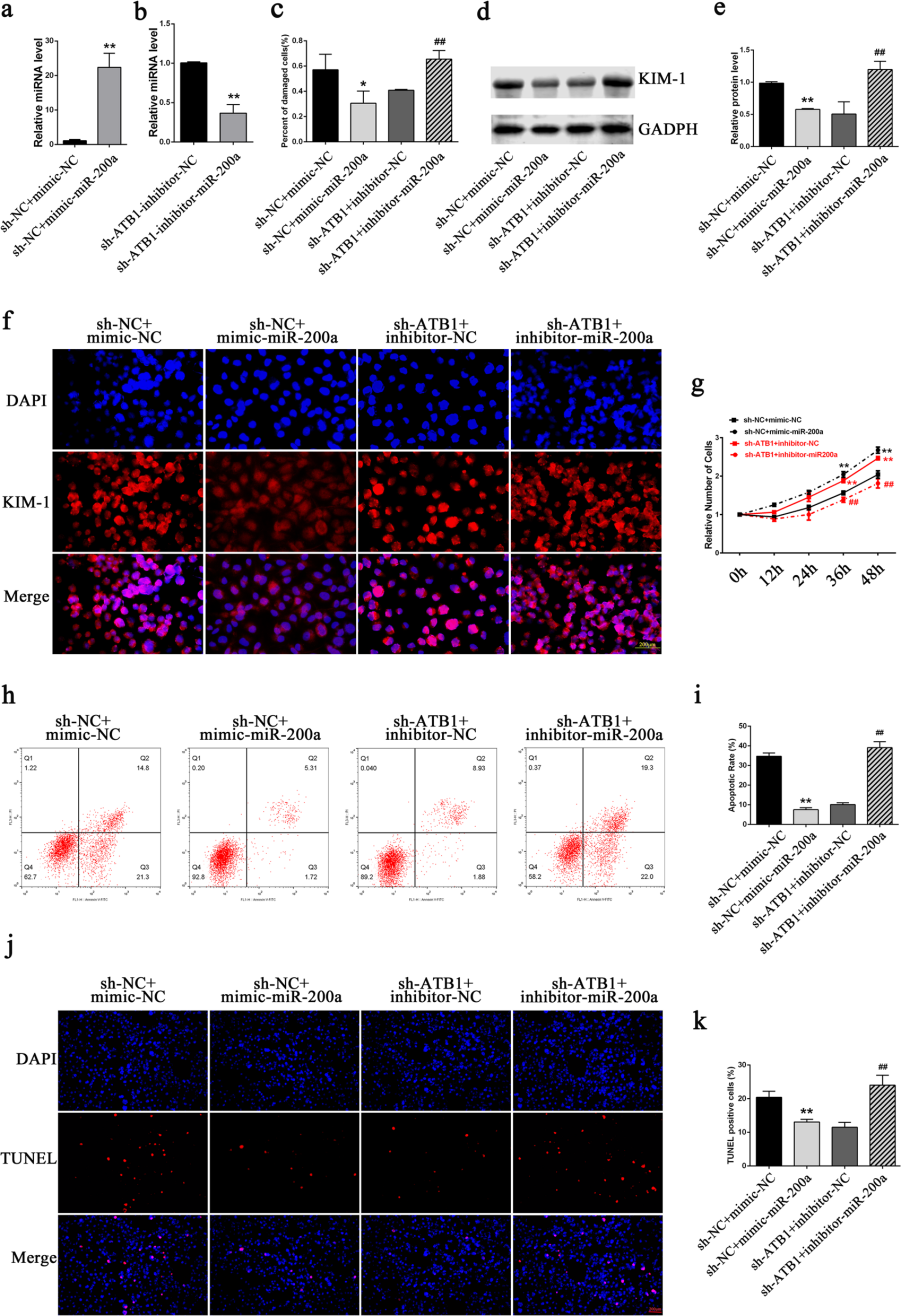
LncRNA-ATB regulates the COM-induced renal injuries via miR-200a. **a**, **b** The transfection efficiencies of miR-200a mimics (**a**) and inhibitors (**b**) were validated by qRT‐PCR. **c** The cell damage was assessed by a Cytotoxicity LDH Assay Kit. **d**, **e** The expression levels of KIM-1 were detected by western blot analysis. **f** The expression levels of KIM-1 were detected by immunofluorescence. **g** Cell proliferation was assayed by CCK-8. **h**–**k** Cell apoptosis measured by flow cytometry **h**, **i** and TUNEL staining (**j**, **k**). All above the experiments were repeated 3 times, values are mean ± SD; *P < 0.05, **P < 0.01 vs. sh-NC + mimic-NC group; ^##^*P* < 0.01 vs. sh-ATB1 + inhibitor-NC group, Scale bars = 200 μm

**Fig. 7:**
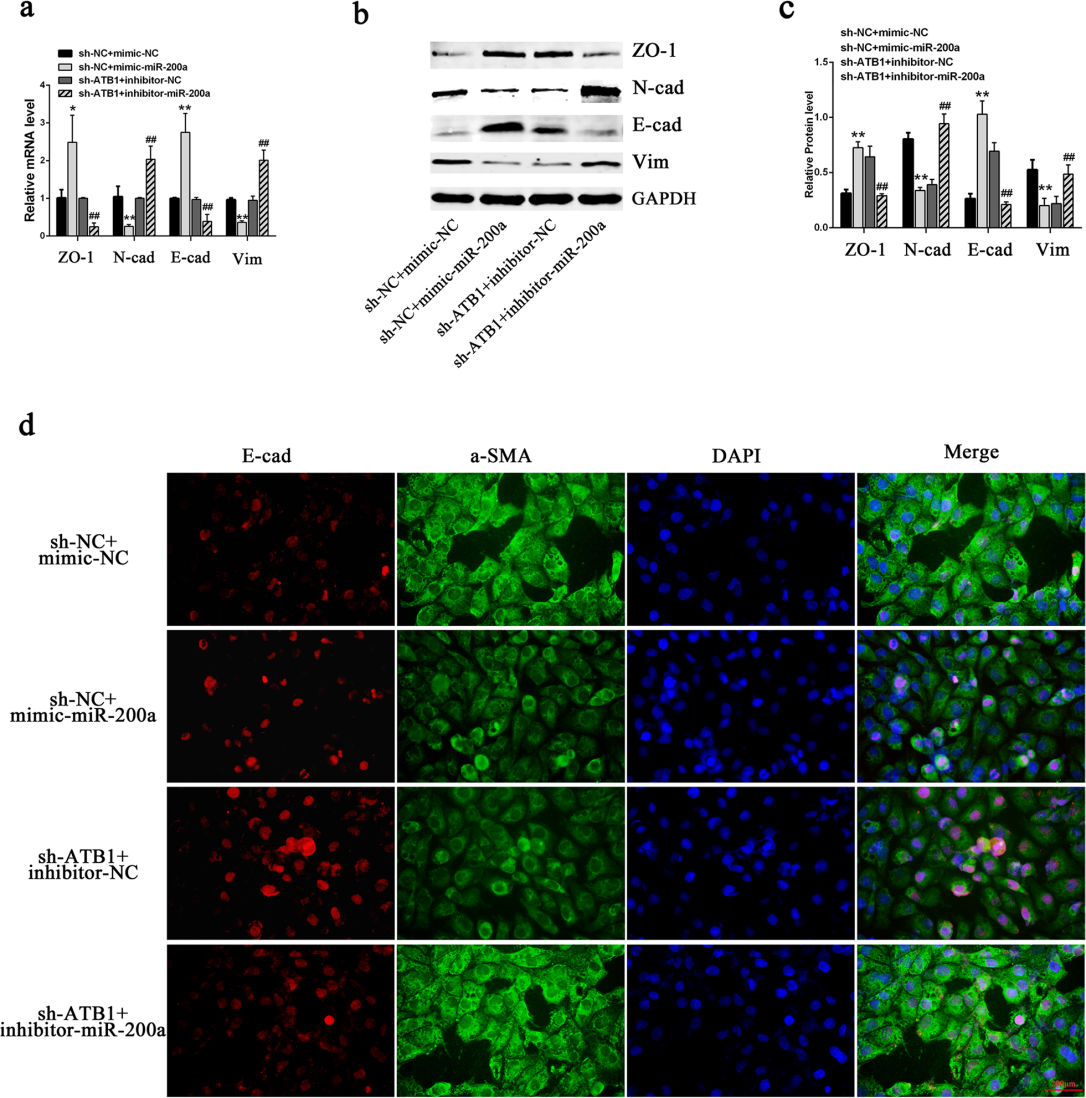
LncRNA-ATB regulates the COM-induced EMT via miR-200a. **a**–**c** The mRNA **a** or protein **b** levels of EMT markers in respective HK-2 cell lines exposed to COM and cotransfected with miR-200a mimics or inhibitors. **c** Quantitative results of EMT marker-protein levels. **d** The expression levels of E-cadherin and α-SMA were detected by immunofluorescence. Scale bars = 200 μm. All above the experiments were repeated 3 times, values are mean ± SD; *P < 0.05, **P < 0.01 VS. sh-NC + mimic-NC group; ^##^*P* < 0.01 VS. sh-ATB1 + inhibitor-NC group

After transfection of the miR-200a mimics into the sh-NC cell line, the miR-200a level in sh-NC cells significantly increased (Fig. 6a). After transfection of the miR-200a inhibitors into the sh-ATB1 cell line, the miR-200a level significantly decreased (Fig. 6b). The LDH and KIM-1 detection results showed that the miR-200a mimics significantly relieved the COM-induced cell injury, whereas the miR-200a inhibitors abolished the relieving effect of lncRNA-ATB interference on this cell injury (Fig. 6c–f). CCK-8 assay results showed that miR-200a relieved the inhibitory effect of COM on cell proliferation, whereas the miR-200a inhibitors aggravated inhibition of cell proliferation (Fig. 6g). Similarly, the detection of apoptosis showed that the miR-200a mimics relieved the COM-induced cell apoptosis, whereas inhibition of miR-200a expression aggravated the COM-induced apoptosis (Fig. 6h–k). The detection of cellular EMT markers showed that miR-200a mimics increased the expression levels of epithelial markers E-cadherin and ZO-1 and decreased the expression levels of mesenchymal markers Vimentin and N-cadherin, whereas the miR-200a inhibitors had the opposite effects (Fig. 7a–c). The same trend was observed in the α-SMA and E-cadherin immunofluorescence assays (Fig. 7d).

## Discussion

Kidney stones are a frequent disease of the urinary system worldwide. The very high incidence rate and recurrence rate after the first treatment still represent a serious health threat and heavy economic burden worldwide (Yasui et al. 2017). The traditional view holds that stones are not harmful to the human body when they do not cause urinary tract obstruction. However, stones have become an independent risk factor for CKD and ESRD (Rule et al. 2009). Crystals are the early stage of kidney stones. Urine in normal people has a large amount of crystal formation. The growth rate of crystals is 1–2 μm/min under supersaturation status, while their retention time in urine in tubules is approximately 5–10 min, and the lumen diameter of renal tubules is 16–60 μm. Therefore, under normal conditions, crystals cannot grow big enough to block the renal tubules. Crystals adhere to renal tubular epithelial cells with a certain probability and stay in the kidney only when renal tubular epithelial cells are injured (Khaskhali et al. 2009).

Persistently high oxalic acid, high calcium or COM stimulation activates nicotinamide adenine dinucleotide phosphate (NADPH) oxidase in renal tubular epithelial cells to produce peroxides and then change mitochondrial permeability transition (MPT), thus causing mitochondrial breakdown (Khan 2013; Yasui et al. 2017). In addition, reactive oxygen species (ROS) are released into the cytoplasm to further aggravate cell injury. Cytochrome *c* released into the cytoplasm can induce apoptosis by activating caspase-9 and caspase-3 (Khan 2013; Yasui et al. 2017). Injured renal tubular cells release many inflammatory cytokines, such as tumour necrosis factor (TNF)-α, TGF-β1, and monocyte chemoattractant protein-1 (MCP-1) to chemotax peripheral monocytes and other inflammatory cells to the injury site. Next, crystals are transferred to the kidney interstitium under the synergistic action of renal tubular epithelial cells and numerous inflammatory cells. Although the activated monocytes/macrophages can phagocytose a small amount of crystals, most of them also badly injured by crystals. Therefore, the crystals cannot be completed cleared (Mulay and Anders 2017). Finally, crystals can only be surrounded to form granulomatous tissues, creating a “nest core” for stone formation, thereby promoting stone formation (Mulay and Anders 2017). In this study, COM stimulation caused significant HK-2 cell injury, inhibited cell proliferation, induced cell apoptosis, and promoted EMT. These results are consistent with many previous study results, including our own (Hu et al. 2015; Li et al. 2019, 2020; Liu et al. 2020a; Peerapen and Thongboonkerd 2020).

Renal tubular epithelial cells in the normal state have a highly differentiated appearance, with a round or polygonal cell morphology. After injury caused by factors such as COM stimulation, renal tubular epithelial cells experience a dedifferentiation process to take on a poorly differentiated appearance with a spindle shape mesenchymal-like phenotype, and their proliferation and migration abilities increase to regenerate tubular epithelium (Zhuang et al. 2005). This process is the so called EMT and belongs to type 2 of the three types of EMT (Kalluri and Weinberg 2009). Since Boonal et al. (Boonla et al. 2011) and Liu et al. (Liu et al. 2013) successively used pathological tissues of stone patients to confirm the involvement of EMT in the occurrence and development of stones, EMT research in the field of kidney stones has gradually received more attention. Stimulation of HK-2 cells by oxalate acid, COM, and TGF-β1 itself all promote TGF-β1 production to promote EMT (Convento et al. 2017; Liu et al. 2020b). Furthermore, renal tubular epithelial cells in kidney stone animal models with hypercalciuria and hyperoxaluria also show obvious EMT changes (He et al. 2015; Liu et al. 2020b). Our previous studies have shown that renal tubular epithelial cells already have EMT changes at the early stage of CaOx crystal-induced renal injury (Hu et al. 2015; Liu et al. 2020a). On one hand, these EMT changes enhance the adhesion ability of tubular epithelial cells to COM to retain crystals in the kidney and thereby promote stone formation (Li et al. 2016, 2019). On the other hand, renal tubular epithelial cell EMT plays an important role in the process of renal interstitial fibrosis and is the critical initiation step in the development of renal fibrosis (Meng et al. 2016; Zeisberg and Neilson 2010). Approximately 30–50% of renal fibroblast cells are from EMT of renal tubular epithelial cells (Zeisberg and Neilson 2010). Renal fibrosis is the common pathophysiological basis of CKD and the eventual ESRD caused by various factors, including kidney stones (Francois and Chatziantoniou 2018). Even though stone obstruction and persistent stimulation can be relieved by surgical measures, these do not halt the process of loss of kidney function and fibrosis (Convento et al. 2017). Therefore, investigation of the mechanism of EMT development and prevention of the occurrence of EMT are key in the prevention and treatment of stone diseases and even of CKD. After coincubation with COM in this study, the morphology of HK-2 cells exhibited an obvious spindle shape (mesenchymal cell morphological change), and the number and length of tentacles increased to exhibit an octopus-like structure. In addition, the epithelial cell markers ZO-1 and E-cadherin significantly decreased, and the mesenchymal markers N-cadherin, vimentin and α-SMA significantly increased.

LncRNAs do not encode proteins. Even so, they have strong biological regulatory functions in both tumour diseases and nontumor diseases. More and more studies in recent years have indicated that lncRNAs also play important roles in the fields of kidney stones and CKD (Song et al. 2019; Zhou et al. 2019; Chen et al. 2020). Our previous studies showed that lncRNA HOXA11-AS regulates inflammatory responses during crystal-induced renal injury through miR-124/MCP-1(Li et al. 2020). LncRNA-ATB is a lncRNA activated by TGF-β. It can regulate the EMT process in liver cancer by competitively sponging the miR-200 family (Yuan et al. 2014). Some studies also showed that TGF-β stimulation significantly increased lncRNA-ATB expression in HK-2 cells and caused significant EMT changes, whereas lncRNA-ATB knockout relieved EMT through different pathways (Qiu et al. 2016; Sun et al. 2020). TGF-β is the most important cytokine in renal inflammation and fibrosis (Tang et al. 2017) and plays an important role in the process of CaOx crystal-induced renal injury (Convento et al. 2017). Therefore, in this study, we selected TGF-β as the positive-control stimulation. The results showed that the TGF-β expression level significantly increased after HK-2 cells were stimulated by COM. LncRNA-ATB in this model was also activated, and its expression level significantly increased. COM and TGF-β stimulation both caused HK-2 cells to undergo significant EMT changes, and interference with lncRNA-ATB expression significantly relieved the EMT changes caused by both; COM-induced cell injury, apoptosis, and proliferation inhibition were also relieved by lncRNA-TAB-interfering. Detection of the expression levels of the miR-200 family in the CaOx cell model showed that all members were significantly downregulated, whereas the expression level of miR-200 s significantly increased in the stably transfected cell lines with lncRNA-ATB interference. In addition, transfection of the miR-200a mimics into COM-stimulated HK-2 cells transfected with sh-NC relieved the COM-induced cell injury, apoptosis, proliferation inhibition, and EMT changes. Transfection of the miR-200a inhibitors into the COM-stimulated sh-ATB1 stably transfected cell line abolished the effects of lncRNA-ATB interference on the relief of cell injury, apoptosis, proliferation, and EMT. Combining previous studies and this study, the results confirm that lncRNA-ATB indeed directly binds miR-200 s. We speculate that lncRNA-ATB exerts the above biological functions through sponging of miR-200 s.

## Conclusion

In summary, lncRNA-ATB had high expression in HK-2 cells injured by COM. It promoted the COM-induced cell injury, cell apoptosis, proliferation inhibition, and EMT and participated in the process of CaOx crystal-induced renal injury by sponging miR-200 s. This study provides new targets and ideas for the prevention and treatment of newly onset and recurrent stone diseases.

## Supplementary Information

**Additional file 1: Table S1. **Primers used for reverse transcription. **Table S2.** Primers used for qRT-PCR.

## Data Availability

The datasets used and/or analyzed during the current study are available from the corresponding author on reasonable request.

## Abbreviations

lncRNA: Long noncoding RNA
TGF-β: Transforming growth factor β
CaOx: Calcium oxalate
EMT: Epithelial-mesenchymal transition
LDH: Lactate dehydrogenase
COM: Calcium oxalate monohydrate
ZO-1: Zonula occludens-1
α-SMA: α-Smooth muscle actin
MiR-200: MicroRNA-200
CKD: Chronic kidney disease
ESRD: End-stage renal disease
ZEB: Zinc finger E-box binding homeobox
Vim: Vimentin
E-cad: E-chadherin
N-cad: N-chadherin
OD: Optical density
TUNEL: TdT-mediated dUTP Nick-End Labeling
KIM-1: Kidney Injury Molecule-1
NADPH: Nicotinamide adenine dinucleotide phosphate
ROS: Reactive oxygen species
MPT: Mitochondrial permeability transition
MCP-1: Monocyte chemoattractant protein-1
TNF-α: Tumour necrosis factor-α
