# Supplementary material for: LncRNA-ATB participates in the regulation of calcium oxalate crystal-induced renal injury by sponging the miR-200 family
Source: Mol Med. 2021 Nov 4;27:143. doi: 10.1186/s10020-021-00403-2 (PMC8567594; doi:10.1186/s10020-021-00403-2)
Supplement: Supplementary file 1 — Additional file 1: Table S1. Primers used for reverse transcription. Table S2. Primers used for qRT-PCR. [file 10020_2021_403_MOESM1_ESM.docx]

Tab. S1 Primers used for reverse transcription

| Names | Primers 5'-3' |
| --- | --- |
| lncRNA-ATB  miR200a  miR200b  miR200c  miR141  miR429 | ACACAGAATAAAATAACAC  GTCGTATCCAGTGCAGGGTCCGAGGTATTCGCACTGGATACGACACATCG  GTCGTATCCAGTGCAGGGTCCGAGGTATTCGCACTGGATACGACTCATCA  GTCGTATCCAGTGCAGGGTCCGAGGTATTCGCACTGGATACGACTCCATC  GTCGTATCCAGTGCAGGGTCCGAGGTATTCGCACTGGATACGACCCATCT  GTCGTATCCAGTGCAGGGTCCGAGGTATTCGCACTGGATACGACACGGTT |

Tab. S2 Primers used for qRT-PCR

| Names | Primers 5'-3' |
| --- | --- |
| lncRNA-ATB(F)  lncRNA-ATB(R)  ZO-1(F)  ZO-1(R)  E-cadherin(F)  E-cadherin(R)  N-cadherin(F)  N-cadherin(R)  Vimentin(F)  Vimentin(R)  GAPDH(F)  GAPDH(R)  U6(F)  U6(R)  miR-200a(F)  miR-200b(F)  miR-200c(F)  miR-141(F)  miR-429(F)  miR-all (R) | TCTGGCTGAGGCTGGTTGAC  ATCTCTGGGTGCTGGTGAAGG  CACGCAGTTACGAGCAAG  TGAAGGTATCAGCGGAGG  GCCCCATCAGGCCTCCGTTT  ACCTTGCCTTCTTTGTCTTTGTTGGA  TGGACCATCACTCGGCTTA  ACACTGGCAAACCTTCACG  CCTGAACCTGAGGGAAACTAA  GCAGAAAGGCACTTGAAAGC  GGAGCGAGATCCCTCCAAAAT  GGCTGTTGTCATACTTCTCATGG  GCTTCGGCAGCACATATACTAAAAT  CGCTTCACGAATTTGCGTGTCAT  CGTAACACTGTCTGGTAACGATG  CGTAATACTGCCTGGTAATGATGA  CGTAAAACTGCCTGGTAATGATG  CGTAACACTGTCTGGTAAAGATGG  CGTACTACTGTCTGGTAAAACCGT  GTGCAGGGTCCGAGGTATTC |
